# Pilot feasibility study of intraprocedural transcranial Doppler monitoring to assess Heal coating thrombogenicity in flow diversion

**DOI:** 10.3389/fneur.2026.1831348

**Published:** 2026-06-24

**Authors:** Anil Arat, Ezgi Yilmaz, Sinan Balci, Ferdi Cay, Charles Matouk, Mehmet Akif Topcuoğlu

**Affiliations:** 1 Department of Neurosurgery, Yale University Medical School, New Haven, CT, United States; 2 Department of Radiology, Hacettepe University Faculty of Medicine, Ankara, Türkiye; 3 Department of Neurology, Hacettepe University Faculty of Medicine, Ankara, Türkiye

**Keywords:** antiplatelet therapy, coating, diffusion weighted magnetic resonance imaging, flow diverter, thrombosis, transcranial Doppler ultrasonography

## Abstract

**Background:**

Utility of flow diverter (FD) surface modification (SM) in reducing thromboembolism of cerebral aneurysm treatment remains debated. Prior studies have relied on clinical outcomes or diffusion-weighted MRI (DWI), which are indirect measures of FD-related thrombogenicity. We used transcranial Doppler ultrasonography (TCD), which enables direct intraprocedural detection of thromboembolic events, to compare the thromboembolic potential of the Derivo2 FD and its coated version.

**Methods:**

A retrospective review of patients monitored noninvasively by TCD during flow diversion was performed in a tertiary center. Due to the variations in physical properties of the FDs and biochemical properties of their SMs, the most commonly used device type was singled out. TCD recordings of 2 patient groups treated with the bare Derivo2 FD (DFD, group 1) versus its coated version (Derivo 2heal FD, D2H) were compared. Group 1 patients were administered dual antiplatelet therapy (DAPT) and group 2 patients were single antiplatelet therapy (SAPT) before the procedure. Groups were compared for emboli (gaseous versus solid) detected continuously starting from the onset of the procedure and extending to 30 min after FD deployment. DWI was obtained within 3 days postoperatively.

**Results:**

13 patients were treated with DFD under DAPT and 13 with D2H under SAPT (consisting of only clopidogrel or prasugrel in 12 patients and aspirin only in one patient). The bare group had more solid emboli within 30 min after deployment (*p* = 0.035) on TCD. When the group 2 patient treated under aspirin only was excluded from the analysis, the significance was marginal (*p* = 0.051). The groups did not differ regarding patient characteristics or DWI hits or antiplatelet therapy response of patients.

**Conclusion:**

In this single-center, hypothesis-generating pilot feasibility study, noninvasive monitoring of thrombi generated during flow diversion was shown to be possible. Although differing antiplatelet regimens across the groups precluded direct comparison, the intraprocedural embolic load in the SAPT-treated D2H group was low, justifying further randomized studies beyond this exploratory study for evaluation of the efficacy of the D2H coating. This study also shows that DWI findings are not reliable specifically for determination of FD thrombogenicity.

## Introduction

Surface modification or coating aims to reduce the thrombogenicity of flow diverters and subsequently decrease the intensity of antiplatelet therapy needed for patients undergoing endovascular aneurysm treatment with these devices (1, 2) but the efficiency of this strategy is still a matter of debate (3). Two randomized studies in relation to the coating of a specific device have recently been completed (4–6) and these studies only demonstrated noninferiority of the coated device by relying on purely clinical data or postprocedure MRI data. We evaluated the efficacy of device coating by utilizing intraprocedural emboli monitoring with advanced transcranial Doppler (TCD) technology, which provides a real-time and a more precise method to detect and characterize microembolic signals as artifactual, solid or gaseous emboli. This method enables direct comparison of the coated and bare devices regarding intraprocedural thromboembolism. We used the number of intraprocedural solid microembolic signals to compare a surface modified flow diverter (Derivo2 FD, with BlueXide surface polish modification, Acandis, Pforzheim, Germany, “DFD”) with its coated counterpart, Derivo 2heal with BlueXide surface polish modification covered by a mesh of fibrin to which heparin is attached covalently (“D2H”) (3). This coating has been developed to decrease the thrombogenicity of the flow diverter, decrease the inflammatory response to it and enhance its endothelialization. However, to date, comparative data to support these assertions have not been available.

## Methods

TCD imaging was initially started in our institution as a post-procedure imaging modality to determine those patients with clinically silent thromboembolic phenomena so that we could adjust the post-procedure antithrombotic medication regimen in these patients. The patients who were amenable to such monitoring had aneurysms of the internal carotid artery and a suitable cranial acoustic window enabling a TCD study. However, this approach proved to be futile after monitoring 25 patients over 2 years on post-operative days 1 to 2, because no embolic signal was detected in continuous 1-h monitoring by a neurologist (MAT) with 25-year experience in neurosonology in these patients. Subsequently, a decision was made to monitor patients during the flow diversion procedure for the next 2 years. Patients who were to undergo an elective flow diversion procedure were initially screened at our TCD laboratory before the procedure to determine whether their transtemporal sonic window is suitable for embolism detection and discrimination. For those patients with a sufficient acoustic bone window, a portable TCD device (Multi-Dop X®, Compumedics Germany GmbH, Singen, Germany) was brought to the angiography suite on the day of the endovascular treatment and the TCD probes were mounted on the patient’s head with a commercial monitoring probe holding head-set (DiaMon®, Compumedics Germany GmbH, Singen, Germany). Detailed description of microembolic signal monitoring and discrimination is presented with examples in the Supplementary file. After administration of general anesthesia, the flow diversion procedure commenced per routine, yet under continuous TCD monitoring. A neurologist (EY or MAT) with experience in emboli monitoring evaluated the signal tracings during the entire procedure and readjusted the probes if needed. The neurologist also noted down the time and duration of the deployment of the flow diverter. As we routinely wait and obtain a final angiographic series 30 min after deployment of any intracranial stent, the monitoring was extended through this 30-min period and then the probes were taken out.

A retrospective review of hospital electronic medical records was undertaken to find those patients who were treated by flow diverters for intracranial aneurysms during TCD monitoring. Technical and logistic factors allowed us to perform intraprocedural TCD monitoring in a total of 36 patients with internal carotid artery (ICA) aneurysms. The TCD imaging was limited to ICA aneurysms as more distal anterior circulation aneurysms and posterior circulation aneurysms cannot be reliably monitored by TCD. Among these patients, 6 were treated with flow diversion and an additional adjunctive device (intrasaccular embolization or stent placement). These 6 patients were excluded from the final analysis. 26 of the remaining patients were treated with either bare or coated Derivo2 line of devices whereas only 4 were treated with other types of coated flow diverters. To achieve homogeneity among the study group with respect to the physical properties of the device, the latter 4 patients were also excluded from the analysis as well, leaving 26 patients treated with bare or coated version of the same type of flow diverter (except for the coating, the physical properties of the bare and coated devices are the same). None of the aneurysms had undergone previous treatment with stents, which would potentially alter the deployment mechanics and thrombogenicity (7).

All of these patients were treated by the senior author under general anesthesia, using a standard triaxial catheter approach consisting of a long sheath, a distal access catheter and a delivery microcatheter. Systemic heparinization was initiated at the beginning of the procedure and reversed routinely after the deployment of the device. As TCD allowed us to have continuous, accurate and instantaneous data about a possible impending thromboembolic complication during the endovascular procedure, we were liberal in offering endovascular treatment under a single antiplatelet agent (APT, clopidogrel 75 mg, starting at least 5 days prior to the procedure). The patients were started on the routine dual antiplatelet regimen (75 mg clopidogrel and 300 mg aspirin daily for at least 5 days). If a patient opted to be treated by a coated flow diverter during the initial pre-procedural TCD screening as an outpatient, the patient was counseled on the possibility of SAPT. If the patient assented, aspirin was discontinued. Thus, patients treated with the bare device remained on a thienopyridine and aspirin whereas those treated with the coated device remained on a thienopyridine only. Platelet function was measured routinely by point of care tests before the procedure and patients who had low response to clopidogrel were switched to prasugrel whereas hyperresponders to prasugrel were switched to a half-dose regimen and reactivity was verified again. Patients underwent a routine non-contrast MRI with diffusion weighted imaging (DWI) before discharge on postoperative days 1 to 3.

IRB approval was obtained for the evaluation of the study cohort. The evaluated data included patient characteristics, platelet response, ipsilateral solid emboli versus gaseous emboli versus artefacts, presence and characteristics of diffusion weighted MRI (DWI) lesions, diameters of the parent artery (proximal and distal landing zones, noted as “afferent artery size” and “efferent artery size”) and aneurysm (dome and neck length) as measured on 2D angiograms.

All data were presented as mean ± standard deviation, or percentage as appropriate. Distribution normality was examined by Shapiro–Wilk’s test. Student’s t or Mann–Whitney U tests were used for numerical values, and chi-square or Fisher’s exact tests were used for categorical variables. Because the data related to the embolic counts and characteristics (gaseous versus solid) measured before/during stent deployment versus after stent deployment for the whole study group were non-normally distributed, the Wilcoxon signed-rank test was used for comparison. A *p* value less than 0.05 was accepted for statistical significance. The relationship between DWI hits and TCD hits was examined using Spearman’s rank correlation coefficient (*ρ*), and statistical significance was assessed using the corresponding *p*-value. SPSS® version 22.0 package statistics program (Armonk, NY: IBM Corp.) was used for all analyses.

## Results

TCD studies obtained on postoperative day 1 or 2 in patients with bare flow diverters did not yield any embolic signals over an imaging period of 60 min and this was in accordance with the only perioperative TCD study related to flow diversion in the literature (8). Intraoperative TCD imaging data was available in 18 female and 8 male patients (mean age 50 ± 14) who were treated for ICA aneurysms. All except one patient had unruptured aneurysms. On pre-procedural TCD evaluation, none of these patients had any hits detected by TCD. A neurosonologist was present for direct monitoring during the whole flow diversion procedure and made sure the recording was diagnostic. If there was inadvertent movement of the probes or signal augmentation due to high flush rates, this neurosonologist immediately alerted the operator and either readjusted the probes or asked the staff to decrease the flush rate to our optimal rate of 1 drop per second. It was not possible to perform thrombotic (solid) MES counts during these masking periods, and therefore we could not provide the timing of the signal loss or interference rate that we anticipated might affect the accuracy of the count. However, the relevant data regarding this effect was anticipated to be equal in both groups given the standard methodology for the interventional procedure and TCD monitoring (including meticulous instantaneous intraprocedural adjustments performed by the neurosonologist).

13 patients were treated with D2H and SAPT (one patient with aspirin only also included in this group), while 13 patients were treated with the DFD and DAPT. Overall, the mean platelet inhibition was 70 ± 15%. 3 patients in the coated group and 2 in the bare group were treated under half-dose prasugrel, otherwise regular doses of these drugs were used. After the evaluation of all embolic TCD signals by the stroke neurologist, TCD data revealed 50 ± 58 solid, 2,325 ± 2,140 gaseous emboli, and 11,251 ± 8,648 artefacts on average ipsilaterally. The afferent and efferent artery diameters of the parent artery were 4.1 ± 0.7 mm and 3.3 ± 0.7 mm, respectively. Aneurysm dome and neck lengths averaged 6.5 ± 2.9 mm and 3.6 ± 1.9 mm. There were no clinically relevant adverse events that occurred perioperatively in this cohort.

An MRI study with DWI was available in 21 patients before discharge from the hospital as part of our postprocedural protocol, in 5 patients MRI was not obtained due to reasons like unavailability of scanner slots in due time or patient unwillingness / claustrophobia. None of these 5 patients had a clinical suggestion of a stroke or TIA. Among the 21 patients, ipsilateral DWI lesions were present in 12 patients (57%). The median number of these DWI lesions was 2 (range: 1–17), and the median diameter of the largest lesion detected in each DWI study was 3 mm (range: 2–13 mm). Of the patients with DWI lesions, the mean age was 54 ± 12 years, and 75% (*n* = 9) were female. Half of these patients (50%, *n* = 6) were treated with the D2H flow diverter. When patients with and without DWI lesions were compared, no statistically significant difference was found in terms of age, sex, type of flow diverter used (D2H vs. bare device), afferent and efferent artery diameters, aneurysm neck size, aneurysm dome size, dome-to-neck ratio, platelet inhibition (%), or flow diverter dimensions (i.e., diameter and length).

One patient in the bare stent group, and another one in the D2H group was switched to prasugrel based on platelet reactivity testing. One patient who had a 2-week history of acute subarachnoid hemorrhage was started only on aspirin with the intention of administering crushed ticagrelor via a nasogastric route if a decision to proceed with flow diversion was made intraprocedurally (that is, after the diagnostic angiogram). A nasogastric tube was placed after the induction of anesthesia and the endovascular procedure commenced without problems. After the flow diverter was deployed, administration of ticagrelor via the nasogastric tube was requested. At that point it was noted that fresh blood was continuously oozing around the nasogastric tube. Anticoagulation was reversed with intravenous protamine sulphate injection. The source of nasopharyngeal bleeding could not be determined by anesthesiology on inspection. As there was no evidence of a thromboembolic events on TCD and angiograms, we decided to delay the administration of ticagrelor. Eighty minutes after the deployment of the flow diverter, the oozing had stopped, there was no evidence of a thromboembolic event on the angiogram and no change in the Doppler spectrum and microembolic signals on TCD study. Based on this finding, Clopidogrel (instead of ticagrelor) was started in a delayed fashion, with a loading dose of 450 mg 4 h after the procedure ended. As the TCD monitoring was performed only during the time this patient was under aspirin only, the patient was included in the SAPT group and the initial 30 min of the post-deployment phase of TCD monitoring was included in the data.

No significant correlation was observed between the number of postprocedural DWI lesions and the number of total emboli detected during the procedure (Spearman’s *ρ* = 0.342, *p* = 0.152), gaseous emboli detected during the procedure (Spearman’s *ρ* = 0.34 *p* = 0.154), or solid emboli detected throughout the procedure (Spearman’s *ρ* = 0.089, *p* = 0.695).

For microembolic signal (MES) determination, inter-reader reliability was assessed in a subset of 7 patients whose MES counts were independently evaluated by two observers (EY and MAT). Agreement was quantified using a two-way random-effects intraclass correlation coefficient (ICC) with absolute agreement. The ICC values demonstrated overall excellent agreement: for ipsilateral solid MES, ICC was 0.954 (95% CI, 0.748–0.992); for ipsilateral gaseous MES, 0.973 (95% CI, 0.865–0.995); for contralateral solid MES, 0.878 (95% CI, 0.440–0.982); and for contralateral gaseous MES, 0.983 (95% CI, 0.903–0.998). Following the demonstration of high inter-reader reliability across measurements, all subsequent MES counts were performed by EY. In cases of uncertainty regarding signal classification, final decisions were reached by consensus between the two neurosonologists.

When the bare and the coated groups were compared for emboli detection after flow diverter deployment, ipsilateral solid emboli counts after deployment did not differ significantly between the groups [median (IQR): 2 (0–5) vs. 2 (0–14); *p* = 0.597], with a small effect size (*r* = 0.14). When the analysis was repeated after transformation to approximate normality, a statistically significant difference was observed (*p* = 0.035). Additionally, the patient treated with the coated device under “ASA only,” as described above, was included in the SAPT arm (coated arm) in this analysis. If this patient is removed from the analysis and comparison is made among patients who received thienopyridines only (alone in the coated arm and together with ASA in the bare arm) there was marginal significance (‘tendency”) regarding this finding (*p* = 0.051). These two separate findings suggested the fragility of statistical significance. The comparison of both groups for the investigated variables is presented in Table 1.

**Table 1:** Comparison of both groups based on the independent variables evaluated in the study.

Variable	Heal and SAPT(*n* = 13)	Bare and DAPT (*n* = 13)	*p*
Age (Mean ± SD)	56 ± 15	45 ± 11	0.098
Female sex (%, *n*)	62% (*n* = 8)	77% (*n* = 10)	0.673
Presence of diffusion lesion (%, *n*)	55% (*n* = 6)	61% (*n* = 6)	1
Ipsilateral solid emboli (Software)	808 ± 682	963 ± 729	0.665
Ipsilateral gaseous emboli (Software)	5,744 ± 8,298	5,376 ± 3,639	0.454
Total emboli (software)	6,553 ± 8,814	6,923 ± 4,376	0.474
Artefact (software)	8,561 ± 5,937	13,343 ± 10,126	0.276
Total HITS (software)	21,821 ± 29,666	22,554 ± 18,456	0.534
Monitoring duration (minutes)	89 ± 20	96 ± 20	0.939
Ipsilateral solid emboli (Before FD deployment)*	35 ± 59	25 ± 41	0.237
Ipsilateral Solid Emboli (during FD deployment)*	12 ± 13	14 ± 18	0.539
Ipsilateral solid emboli (after FD deployment)*	4 ± 6	10 ± 15	**0.035**
Ipsilateral total solid emboli*	51 ± 67	48 ± 50	0.402
Ipsilateral total gaseous emboli*	1751 ± 1,693	2,899 ± 2,441	0.243
Total solid emboli (right and left) *	53 ± 72	55 ± 53	0.373
Aneurysm neck (mm)	4 ± 2	3.1 ± 1.7	0.507
Aneurysm size (mm)	6.9 ± 3.4	6 ± 2.3	0.326
Dome to neck ratio	2 ± 1.1	2.2 ± 1	0.424
Platelet function test (Efficiency %)	64 ± 12	75 ± 17	0.480
Number of ipsilateral DWI lesions	4 ± 6	3 ± 5	0.355
Size of the largest ipsilateral DWI lesion (mm)	8 ± 4	3 ± 3	0.393
Flow diverter diameter (mm)	4.6 ± 0.5	4 ± 0.8	0.198
Flow diverter length (mm)	28.5 ± 10.7	23.9 ± 9.2	0.415

Heal, group treated by Derivo 2heal device; Bare, group treated by the bare device; SAPT, single antiplatelet agent (includes one patient treated under aspirin only); DAPT, double antiplatelet agent; Ipsilateral, on the side of the placement of the flow diverter; Software, emboli detected by the automated detection software of the TCD system; HITS, total number of embolic signals, *Number of emboli as determined by evaluation of signal characteristics of each embolic signal of the TCD study by stroke neurologist; DWI, diffusion weighted magnetic resonance imaging. *P* values with significance are written in bold.

When the whole cohort was evaluated, on the side of flow diversion, significantly lower embolic counts were noted after FD deployment as compared to before/during FD deployment. This was valid for both solid and gaseous emboli (*p* < 0.001 for both, Table 2).

**Table 2:** Comparison of number of emboli before/during device deployment versus after device deployment.

Ipsilateral Emboli	Before and during deployment Mean ± SD	Before and during deployment Median (IQR)	After deployment Mean ± SD	After deployment Median (IQR)	*Z* value	*p* value
Ipsilateral solid emboli	43 ± 56	18 (5–79)	7 ± 12	2 (0–11)	−3.636	<0.001
Ipsilateral gaseous emboli	1824 ± 1740	1,385 (763–2021)	501 ± 868	208 (74–460)	−4.254	<0.001
Ipsilateral total emboli	1867 ± 1732	1,451 (858–2,118)	507 ± 872	210 (77–468)	−4.254	<0.001

SD: standard deviation; IQR, interquartile range.

In multivariable linear regression analysis, using log-transformed post-deployment ipsilateral solid MES counts as the dependent variable, no independent predictors of embolic burden were identified. Procedure duration (*β* = −0.098, *p* = 0.655), afferent artery diameter (*β* = −0.367, *p* = 0.093), aneurysm neck width (*β* = 0.096, *p* = 0.708), and flow diverter length (*β* = 0.166, *p* = 0.509) were not significantly associated with MES counts.

## Discussion

Currently the 4 types of “coated” flow diverters available for use are Pipeline Shield (Medtronic, Irvine, CA), Phenox HPC (Bochum, Germany), FRED X (TerumoNeuro, Aliso Viejo, CA) and D2H (9, 10). Of these devices, the most widely studied is the Pipeline Shield with phosphorylcholine surface modification. Different forms of this modification (dopamine-phosphorylcholine) have been proven safe without a need for any APT in animal models (11). Yet, the claim that of the Pipeline device lowers the rate of thrombotic complications has not been ascertained. In a large and matched series by El- Naamani et al., the surface modification did not result in decreased thromboembolic complications or better occlusion rates (12). Similarly, in an even larger, propensity matched cohort of 707 patients studied specifically for thromboembolic complications (TE), Ramirez-Velandia et al. (13) found no difference between the modified and bare Pipeline device. Lastly, both types of devices were similar with respect to perioperative diffusion-weighted MRI findings in another study (2).

The second device with more abundant literature data is the p64/48 family of flow diverters. To compare these flow diverters with the bare device, Bilgin et al. performed a meta-analysis (1). They found that the ischemic complication rate for HPC to be 7.3%. The bare device had a rate of 5.3% and the difference was not significant.

The only comparison of the FRED-X versus the bare FRED device from a clearly defined thromboembolic standpoint has been published by Goertz et al. (14) and failed to show a significant difference in periprocedural stroke rate between the bare and coated device. Although these authors mentioned about a “tendency” toward significance (*p* = 0.07) after multivariate analysis, the generalizability of this finding is not straightforward for the following reasons: (1) The perioperative stroke rate in the bare arm of this specific study (5.1%) is higher than the rate in the largest and/or most recent multi-center studies (1.6% in 579 aneurysms, EuFRED Study and 0% in the FRED-UK Study) (15, 16) or meta-analysis studies (4%) (17). (2) the perioperative risk of stroke in the FRED X arm is lower than the 5% risk reported in both the unruptured cohort of the initial US multi-center study (18) as well as the US multicenter post-market study of FRED X in unruptured aneurysms (19). That is to say the participating centers of the study by Goertz et al. had exceptional results in the FRED X arm that may not generalized to the general endovascular practice. Despite these biases, a significant benefit for TE events was not demonstrated for FRED X in this study. A second and very recent study claiming to compare both devices found a significant difference, favoring the coated device (20). However, the evaluation criteria in this retrospective study appears to be structured in a way that benefits the coated device due to the following reasons: (1) Over 9 years, between 2013 to 2022, 19 bare devices were used by these authors despite the exclusive use of 37 coated devices in the following 3 years suggesting a bias secondary to the learning curve effect. (2) Preprocedural PRU was significantly lower in their coated group, not only showing a definite difference between the groups, but suggesting perfection of technique and other procedural criteria for flow diversion between 2013 to 2022. (3) Jailed branch thrombosis (a.k.a. “Coanda Effect”), a hemodynamic phenomenon, was included as “thrombotic event.” (4) a composite endpoint of “DWI abnormalities plus thrombotic events” was tailored specifically for evaluating the results. Apart from this latter study, there is also a large-scale comparative study on FRED and FRED X published by Guimaraens et al. (21), but unfortunately, the exact nature or timing of thromboembolic complications were not given separately for each device type in this study.

Lastly, there is one very recent study comparing TE complications of bare versus coated devices of the Derivo device line in the literature (22). Regrettably, this study has only 21 patients, in whom the unruptured aneurysms were treated mainly (63%) with the coated device whereas 80% of the acutely ruptured aneurysms were treated with the uncoated device. The outcome was similar in both groups. Due to the very limited number of patients and clinical discrepancies in the cohort, the authors accepted that there is a bias which prohibits conclusions about device thrombogenicity based on their data. Randomized data for head-to-head comparison of bare and coated devices is not currently available.

Nonetheless, randomized prospective multicenter data is available regarding SAPT utilization for coated flow diverters (DART study 5). Since coatings are unique in biologic properties for each device, the results are limited to the HPC coating. Our study stands out as the only currently available study about the embolic profile of flow diverter devices during and after deployment. In the meantime, it also provides data unique to the HEAL coating. The first and possibly most important suggestion of this study is that the embolic potential of bare flow diverters in patients under appropriate APT becomes negligible immediately after deployment and the embolic potential practically disappears as of postoperative days 1 to 2. The second suggestion is, the majority of procedural emboli occur before or during device deployment and are thus related to technical factors. Thirdly, there is no strong relation between the emboli generated during the procedure (i.e., embolic “hits” versus DWI ‘hits”). This is not unexpected as most of the potentially dangerous emboli to the brain are either overcome by the collateral circulation of the brain or they are transient in nature. This underscores the superiority of TCD as compared to DWI for evaluation of acute thrombogenicity of specific devices. However it is critical to note that device thrombogenicity is only a part of the overall clinical safety of a flow diverter or, in general, flow diversion. TCD is a good indicator of acute device thrombogenicity rather than overall clinical safety whereas DWI is known as a reliable indicator for overall neural tissue damage and has been utilized in randomized studies evaluating FD coatings, such as the COATING study (CS) (4) but does not have the specificity to determine only device-specific thrombogenicity. In other words, DWI is an indirect and imperfect surrogate marker for device thrombogenicity but a reliable one to determine the “net thromboembolic consequence” of a flow diversion procedure, remaining after all embolic events generated during the procedure are counteracted by cerebral defense mechanisms (e.g., dissolution of clot, collateral circulation) that have occurred from the onset of the procedure to the time of the postoperative MRI scan. Our findings by no means refute the role of postoperative role of DWI for procedural evaluation, which matters most in patient care. It is rather a methodological complement that can determine the contribution of the device-induced acute thrombi to the final outcome. We demonstrated that not all the emboli result in DWI hits (all patients had emboli but some did not have DWI hits) and not all the emboli are caused by the flow diverter (actually, majority of the TCD hits, were not). Thus, as previously suggested (3), the conclusions of the current studies that utilize DWI as an endpoint [i.e., CS study claiming noninferiority of HPC coating or the study by Ziegenfuss et al. (23)] claiming futility of the coatings remain speculative rather than decisive about the thrombotic profile of specific devices. Notwithstanding their seemingly contradictory results, these studies converge on the same conclusion that coated and bare devices have comparable safety under dual antiplatelet therapy. Our study actually suggests an advantage of a specific flow diverter coating regarding thrombogenicity based on direct emboli monitoring. However, our suggestions remain suggestive rather than conclusive due to the limited number of patients, which is the main limitation of the study, and our findings apply exclusively to the HEAL coating.

Last but not least, when SAPT is defined as prasugrel or clopidogrel only (either regular or half-dose regimen) there was marginal statistical significance between the bare and coated groups. When SAPT is defined as “any single antiplatelet medication,” the patient treated under only ASA gets included in the SAPT group. Then, a significant difference among groups is noted, implying the superiority of the coated group in terms of thrombogenicity. One of our patients had to be bridged with only orally administered aspirin and showed minimal intraprocedural embolic load. Anectodal flow diversion cases under ASA only has been reported for D2H (24), after IV aspirin administration in contrast to our case. We suggest that, if necessary, such anectodal applications should be performed under advanced imaging such as TCD monitoring or optical coherence imaging due to the high risk of stent thrombosis.

When evaluating SAPT for coated FD, it is critical to mention that randomized evidence about the safety of SAPT for *bare* devices is still lacking and this has remained so after the CS study (6) and the DART study (5). The second randomized study, namely, the DART study did not include a bare arm at all. In our opinion, randomized data about SAPT for bare devices is critical to have because, the standard of using clopidogrel with aspirin has origins dating to the earlier times of intracranial stenting when antiplatelet resistance was not routinely measured. In such a setting, such a “blanket” strategy was very reasonable and has proven to be safe for more than two decades since the resistance to aspirin and clopidogrel rely on separate biologic pathways. A recent meta-analysis by Senol et al. showed that the risk of ischemic complications when FDs are placed under SAPT is around 21 and 6% for aspirin and clopidogrel, respectively (25). Given these rates, the practice of using both clopidogrel and ASA simultaneously lowers the risk of overall platelet resistance as pathways for resistance to these medications are not directly related to each other. That is to say, low or no response to clopidogrel may be counterbalanced by a response to ASA. We believe that when thienopyridines are used as SAPT *after* reactivity testing, it stands to reason to replace DAPT by SAPT with new generation thienopyridines (rather than ASA only) for all kinds of flow diverters or intracranial stents regardless of the coating. This claim is supported by other studies as well (26).

To say it in other words, the current DAPT regimen, and for that matter, the current platelet reactivity threshold, may have been set high enough to cover a variety of thromboembolic causes such as malapposition, surface irregularity/non-uniformity, device configuration problems (fishmouthing, bottle-neck formation) and individual responses to stent materials. In such a multi-factorial environment, demonstration of a possible positive effect of device coating on thromboembolism, merely based on clinical outcome or DWI appears to be a far-fetched aim. Therefore, FD coatings should be investigated for added benefit in patients with higher baseline platelet reactivity (e.g., with reduced dose therapy) or with increased likelihood of perioperative thrombogenicity (i.e., acute subarachnoid hemorrhage) or relative contraindications to DAPT (27) by state-of-the-art imaging such as TCD or OCT (28), beyond plain MRI or clinical findings. Our study suggests that HEAL coating warrants further evaluation in randomized and/or multicenter studies for patients who have higher baseline platelet reactivity/thromboembolic risk.

In our experience, the main limitations of intraoperative TCD for flow diversion were logistic constraints, and some shielding of the target arterial segment by TCD transducers in the working projection during endovascular treatment. The ability to monitor only internal carotid artery aneurysms in patients with suitable acoustic windows limits the generalizability of the findings, without diminishing the validity for the selected sample, the main target population of flow diversion. Increased streaking artefacts on post-procedure flat panel CT may also be a concern. Again, in our experience, background Doppler signal augmentation during angiographic runs and when the guiding catheters are flushed briskly may conceal gaseous or solid emboli. We noticed that angiographic runs frequently result in a burst of microembolic signals that is beyond the automated characterization sensitivity of the device. Yet, this effect is expected to be similar in both cohorts and TCD was a reliable imaging modality for intraoperative monitoring with a high inter-reader reliability. In addition to these technical limitations, as noted above, our study is also limited by its retrospective design and limited number of patients leading to fragility of the statistical findings. Moreover, our results are valid for the period that encompasses the period between the onset of the procedure and postoperative day 3. It is conceivable that thromboembolic events can occur beyond that period and this is relevant especially to patients who are treated under ASA only. Delayed stent thrombosis is known to occur in those patients (29). Finally, it can be argued that TCD hits in totally asymptomatic patients cannot be a surrogate of device safety from a clinical perspective. These hits have recently been shown to be associated with worsened long-term cognitive function (27).

## Conclusion

There is no previous data about the measurement of thromboembolic events intraoperatively and in a hyperacute fashion during flow diversion. As the pilot feasibility study regarding intraprocedural TCD monitoring for flow diversion, our study fills the knowledge gap regarding the thromboembolic profile of flow diversion intraprocedurally/during hyperacute phase. Our study shows that solid emboli after device deployment is significantly less in appropriately premedicated patients and in such patients, the postprocedure DWI abnormalities are not specific markers for device thrombogenicity. Although the device coating cannot be singled out as the direct cause of the TCD findings due to the differences of APT in both groups (which is similar to the APT allocation in the COATING study) we demonstrated by direct emboli imaging, that D2H device deployment under the lower-intensity APT regimen (SAPT) did not result in a higher intraprocedural embolic load as compared to the bare device deployment under higher intensity APT regimen (DAPT). As a hypothesis generating report, the current exploratory study signals a possible advantage of D2H coating which should be comparatively evaluated in larger cohorts utilizing the same type of APT, preferably SAPT, for both type of devices.

With the widespread use of ticagrelor and prasugrel based regimens, we should shift our attention to the level of platelet inhibition rather than the number of medications to attain the inhibition. The high efficacy of these two drugs may be masking a positive effect of device coating on thromboembolism. Hence flow diverter coatings should be further investigated for added benefit in patients with higher baseline platelet reactivity or with increased perioperative thrombogenicity (i.e., acute subarachnoid hemorrhage) as documented by platelet reactivity testing and as demonstrated by state-of-the-art imaging such as TCD or OCT in the context of controlled prospective trials.

## Data Availability

The raw data supporting the conclusions of this article will be made available by the authors, without undue reservation.
