# Supplementary material for: Pilot feasibility study of intraprocedural transcranial Doppler monitoring to assess Heal coating thrombogenicity in flow diversion
Source: Front Neurol. 2026 Jun 24;17:1831348. doi: 10.3389/fneur.2026.1831348 (PMC13341477; doi:10.3389/fneur.2026.1831348)
Supplement: Supplementary file 1 [file Data_Sheet_1.PDF]

Supplementary Methods

Transcranial Embolic Signal detection, Discrimination and Analysis

Setting: After endotracheal intubation in the angiography suite, bilateral Transcranial Doppler (TCD) monitoring probes were placed for continuous microembolic signal recording. Each patient underwent an average monitoring duration of 93 ± 20 minutes, including a standardized 30-minute period following stent deployment. Bilateral 2.0 + 2.5 MHz multifrequency probes were used with a headset (DiaMon®, Compumedics Germany GmbH, Singen, Germany) for secure positioning. The ultrasound system utilized was the DWL MultiDop X® model, and off-line data analysis was performed using DWL QL® software (Both, Compumedics Germany GmbH, Singen, Germany). The microemboli differentiation transducers were larger in size due to their multifrequency capability, yet their dimensions did not interfere with procedural workflow. (Figure 1)

Ultrasound Signal Acquisition and Analysis: Monitoring was performed bilaterally, covering both the operative and non-operative sides, with insonation targeted at the proximal segment of the middle cerebral artery (MCA). This segment is located distal to the aneurysm treatment area. All microembolic signal (MES) analyses were conducted offline and were performed blinded to the type of stent used.

Microembolic signal detection and discrimination was performed in two stages. In the first stage, all high signal events (HITS: High Intensity Transient Signal), determined by the DWL embolism detection and discrimination program (DWL QL®), were counted. An example is marked as an "event" in Figure 2. These signals are diagnosed by the software as “artefact”, “solid embolus” or “gaseous embolus” (Box in the right bottom).

In the second stage, all of these signals (including artefacts) were reviewed separately by two sonographers (YY and XXX). The solid embolus numbers and signals were then compared and the final number was determined by consensus. An inter-reader ratio was not calculated. Each signal was reclassified as "Solid" or "Gaseous" based on the diagnostic category of the software, the duration of the embolic signal, MEBR (measured embolism-blood ratio), frequency modulation, flow modulation and acoustic properties such as compressibility.

During analysis, we observed that signal artifacts—especially those caused by saline flushing, contrast agent injection, aliasing, and device manipulation—frequently altered the MEBR, leading to misclassification by the automated DWL QL® software. In particular, the software occasionally misidentified artifacts as microembolic signals (MES) and demonstrated limited reliability in differentiating solid vs. gaseous emboli under these conditions. (Figure 3) To address this, all HITS automatically detected by the software were visually reviewed by evaluating their spectrogram appearance, raw Doppler signal, and M-mode traces (Figure 4a-c).

Solid and gaseous emboli were viasually classified and counted in 5-minute epochs. On average, 22233 ± 23105 HITS were analyzed per patient.

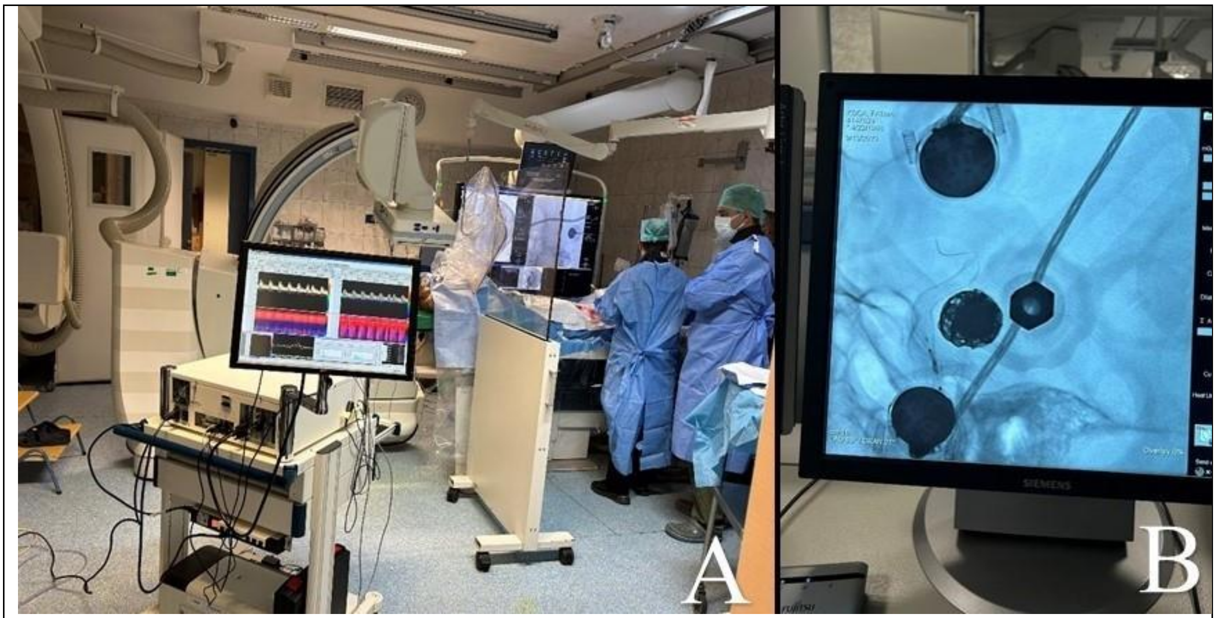

Figure 1. (A) General view of the angiography suite during transcranial Doppler (TCD) monitoring, showing the TCD system interface and the procedural setup. (B) Fluoroscopic image displaying the positioning of the transducers in relation to the aneurysm and the deployed flow diverter stent.

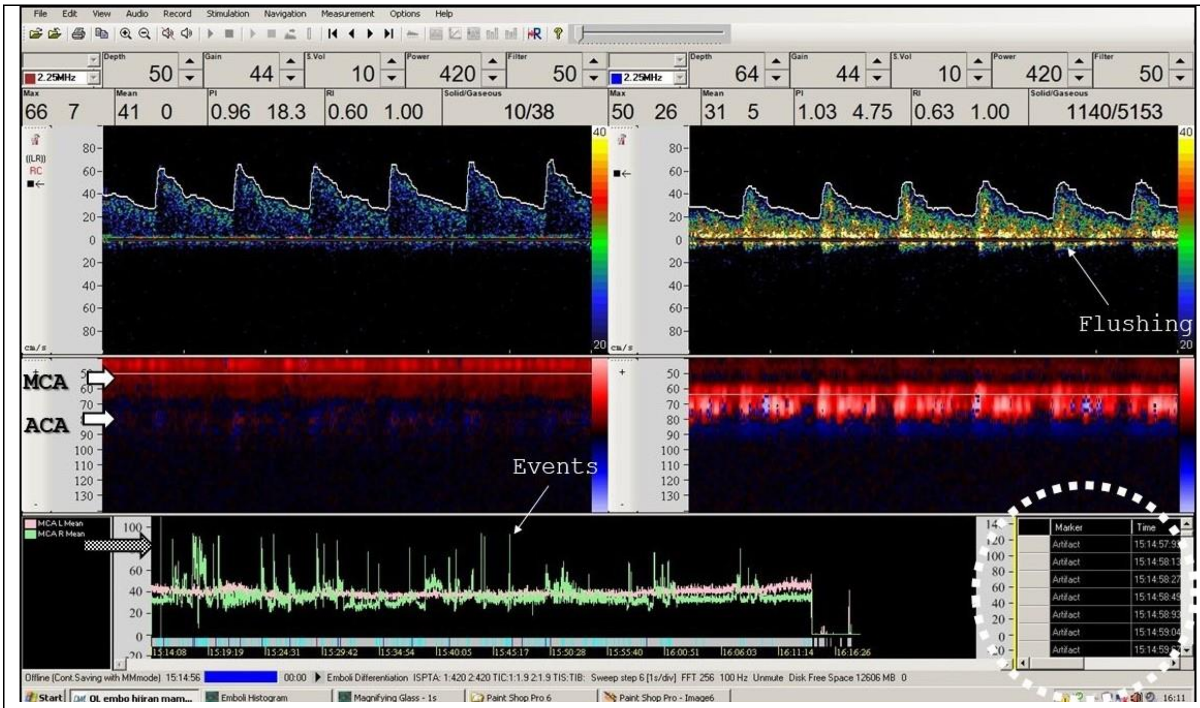

Figure 2. DWL QL® software interface showing M-mode visualization of ACA (blue, away, anterior cerebral artery) and MCA (red, towards, middle cerebral artery) waveforms. Detected events are indicated in two ways: (1) along the event timeline bar at the bottom of the screen, marked by a checkered arrow, and (2) in the event classification panel at the lower right corner, enclosed within a dashed round marker. The software automatically categorizes signals into artifacts, solid emboli, and gaseous emboli, based on their acoustic and sonomorphological features.

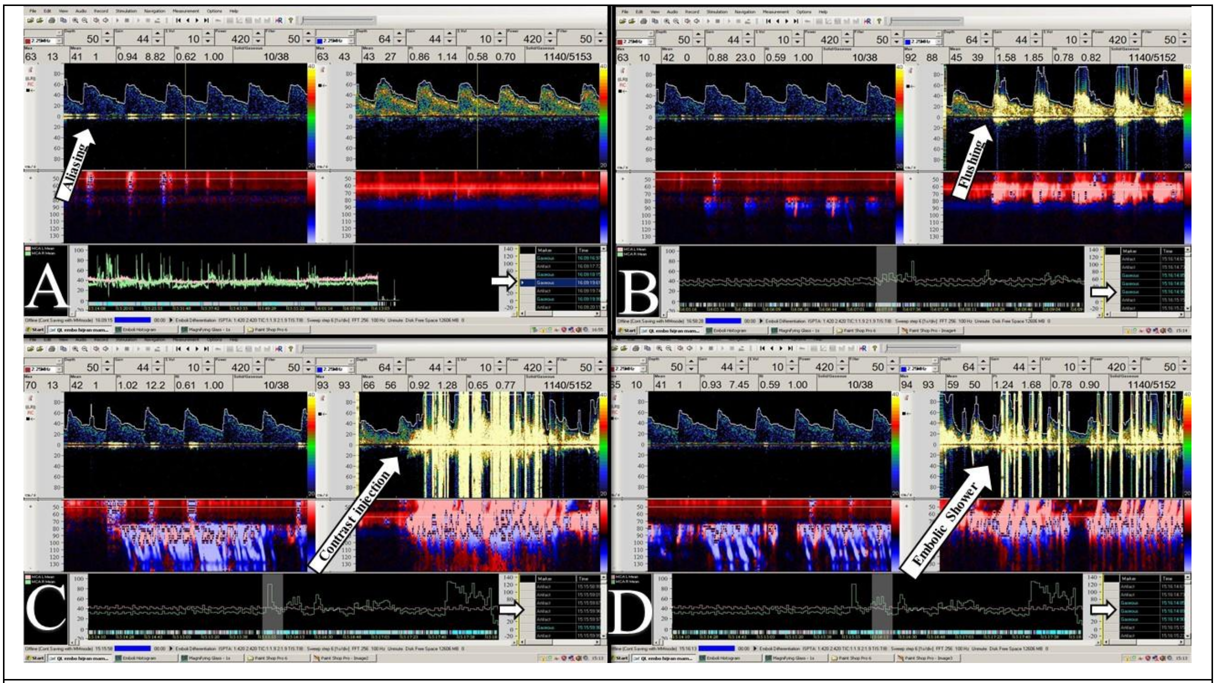

Figure 3. Representative screenshots from DWL QL® software illustrating common scenarios that interfered with accurate MES detection and differentiation, ultimately necessitating manual embolic signal classification.

(A) Aliasing artifact mimicking MES signals, leading to false positives.

(B) Failure of the software to distinguish MES during saline flushing, likely due to transient changes in the measured embolus-to-blood ratio (MEBR).

(C) Inability to accurately classify embolic signals during contrast agent injection, also associated with MEBR shifts.

(D) Loss of MES differentiation during an embolus shower immediately following stent deployment.

In all panels, misclassified embolic signals and their corresponding spectrogram features are highlighted with arrows.

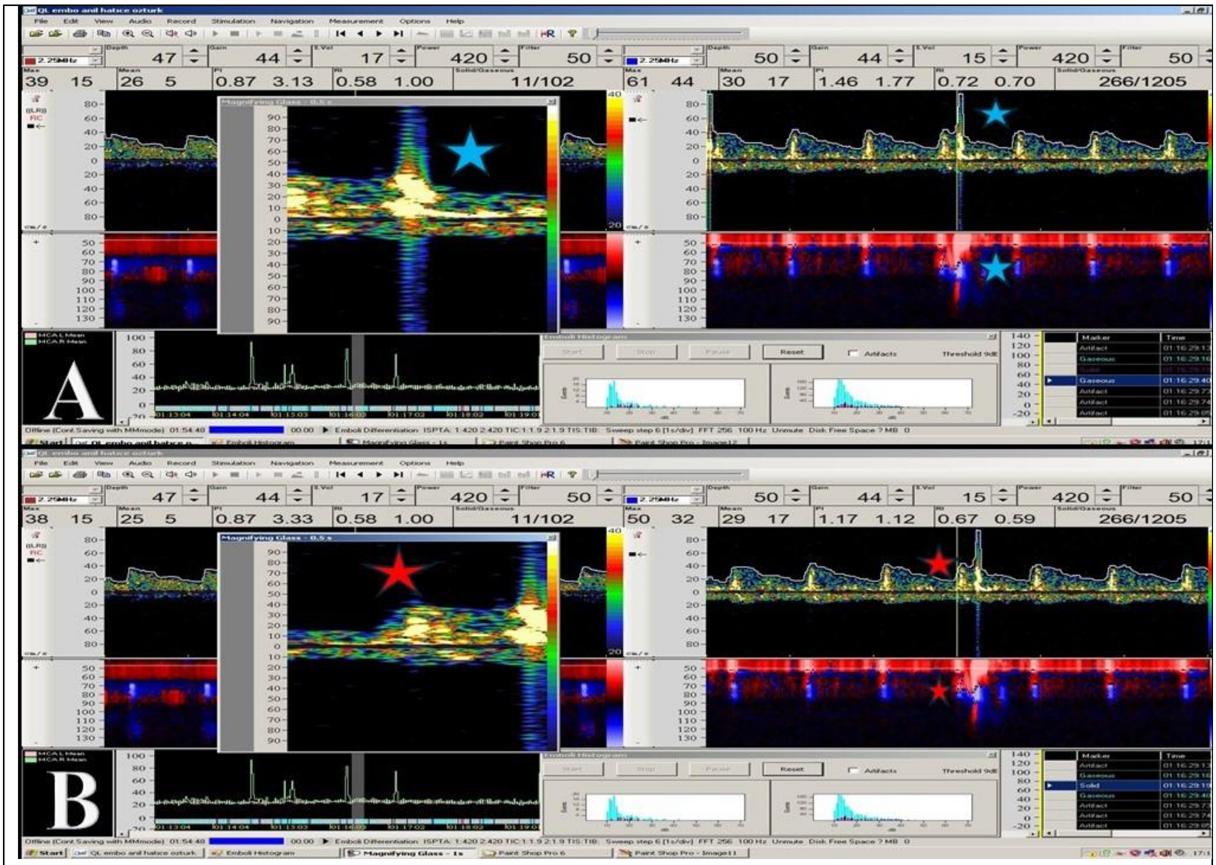

Figure 4a. Examples of typical solid and gaseous MES signals. A and B : The spectrogram and M-mode images of a gas and a solid embolus. The gas embolus is marked with a blue star, and the solid embolus with a red star

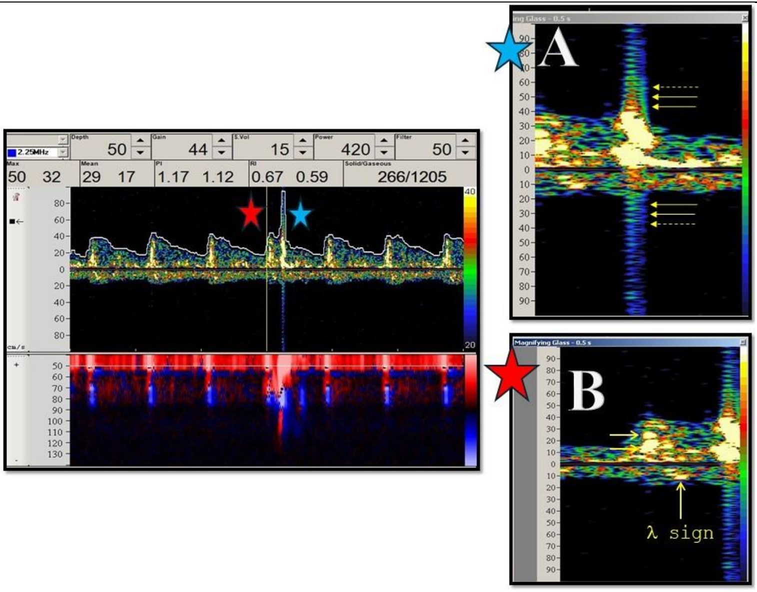

Figure 4b. Sonogram characteristics of solid [Red star, B] and gaseous [Blue star, A] MES signals depicted in same cardiac cycle. The zoomed-in spectrogram shows the typical 'lambda' or 'tail sign' for the solid embolus, highlighted within open arrows. Infinite reverberation effect (parallel increasingly contracting lines) is characteristics of pure solid embolic signal (closed arrows).

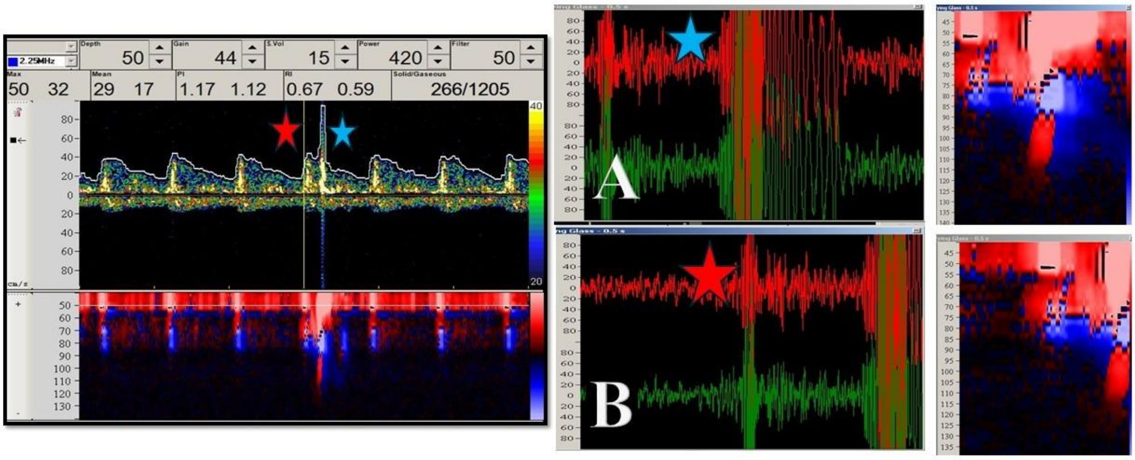

Figure 4c. Raw Doppler signal and zoomed-in M-mode trajectory images (center and right side, respectively). Gaseous MES (marked with blue asterisks) are characterized by typically high MEBR, accordion sign (indicator of frequency modulation), and echo transition (anechoic rim in trajectory M-mode), whereas solid MES (marked with red asterisks). The slope of embolic propagation trajectories is highlighted in the zoomed-in M-mode image.
